# A Study on the Surface Oxidation Pretreatment and Nickel Plating Mechanism of Carbon Fiber

**DOI:** 10.3390/ma17153650

**Published:** 2024-07-24

**Authors:** Qinghui Wang, Xuesong Li, Dongdong Zhu

**Affiliations:** 1 Key Laboratory of Advanced Structural Materials of Ministry of Education, Changchun University of Technology, No. 2055 Street Yanan, Chaoyang District, Changchun 130012, China; wangmian203@126.com (Q.W.); 2202102070@stu.ccut.edu.cn (D.Z.); 2 School of Mechanical Engineering, Changchun Technical University of Automobile, No. 1777 New Hongqi Street, Luyuan District, Changchun 130013, China

**Keywords:** carbon fiber, surface modification, functional group, electrodeposition

## Abstract

This study explores the effects of various temperatures on the surface modification of carbon fibers, as well as the effect of differing voltages and currents on the morphology, deposition rate, and thickness of the Ni plating layers. Post-treatment characterization of the samples was conducted utilizing scanning electron microscopy (SEM) and X-ray photoelectron spectroscopy (XPS) methods, thus facilitating a discussion on the mechanism of Ni plating. The findings demonstrate that at a temperature of 500 °C, the carbon fiber surface exhibits the highest concentration of functional groups, including hydroxyl (-OH), carboxyl (-COOH), and carbonyl (-C=O), resulting in the most efficacious modification. Specifically, exceeding 500 °C leads to significant carbon fiber mass loss, compromising the reinforcement effect. Under a stable voltage of 7.5 V, the Ni-plated layer on the carbon fibers appear smooth, fine, uniform, and complete. Conversely, at a voltage of 15 V, the instantaneous high voltage induces the continuous growth of Ni^2+^ ions along a singular deposition point, forming a spherical Ni-plated layer. In addition, a current of 0.6 A yields a comparatively uniform and dense carbon fiber coating. Nickel-plated layers on a carbon fiber surface with different morphologies have certain innovative significance for the structural design of composite reinforcements.

## 1. Introduction

Carbon fiber, a cutting-edge material composed of over 95% carbon, exhibits exceptional properties, including high tensile strength, a high modulus, and remarkable resistance to thermal shock, fatigue, and acidic environments [1,2]. These attributes make it a highly sought-after reinforcement material for the fabrication of both metal- and non-metal-based composites. However, commercially available carbon fibers possess inherently smooth surfaces with a low surface energy and high inertness, lacking chemically active functional groups [3,4]. This characteristic leads to poor interfacial wetting in composite materials, hindering the formation of robust and effective interfacial bonding and thereby limiting their broader applicability in carbon fiber composites. To address this challenge, surface modification of carbon fibers emerges as a critical step in achieving high-performance carbon fiber composites. Carbon fiber surface treatment methods mainly fall into two categories, physical and chemical methods, among which physical methods are mainly improved by increasing the specific surface area and roughness so as to improve the physical adhesion of the interface between carbon fiber and resin, such as heat treatment coating methods, plasma modification, etc. [5,6]. Chemical methods increase the chemical functional groups on the surface of the carbon fiber and improve the method of chemical bonding to increase the bond strength of the interphase of the composite material, such as chemical grafting modification, plasma modification, and metal modification of the surface of carbon fiber [7,8,9]. Among various modification methods, the metallization of carbon fiber surfaces has captivated significant interest due to its promising potential [10,11,12]. By depositing different metals, alloys, or composite layers onto the surface of carbon fibers, the advantages of the metals can be effectively combined with the exceptional properties of carbon fibers. This approach not only enhances the wettability between the carbon fibers and metals or resins, reducing interfacial reaction issues, but also facilitates the development of carbon-fiber-reinforced composite materials with superior overall performance [13,14,15].

The traditional activation method of electroless nickel plating has high requirements and complex mechanisms, and there are few kinds of activators available for electroless nickel plating, which increases the difficulty of the research. Ni electroplating on carbon fiber surfaces represents a potent method for enhancing their wettability. This surface treatment method boasts several advantages, including facile equipment requirements, rapid plating speeds, cost-effectiveness, and minimal environmental impact. Specifically, Ni exhibits an excellent bonding affinity with carbon fibers [16]. And the weight of nickel is relatively light, and the quality of nickel layer is relatively stable, so the surface metallization of carbon fiber mostly involves treatment using nickel plating. This study evaluated the effects of high-temperature oxidation modification and Ni plating processes on carbon fiber surfaces. Specifically, the research evaluated the effect of the oxidation temperature on surface modification and the density of the functional groups on the carbon fibers. Furthermore, the study explored the effect of voltage and current intensity on the morphology and thickness of the Ni-plated layers. Finally, the research explained the formation mechanism of carbon fiber coatings under varying morphologies.

## 2. Materials and Methods

### 2.1. Experimental Materials

The experiments employed polyacrylonitrile (PAN)-based 3K-T300 carbon fibers, characterized by a filament count of approximately 3000 per bundle. These fibers possess advantageous properties including a low density, high specific strength, and high specific modulus. The detailed chemical and physical characteristics of the utilized carbon fibers are presented in Table 1.

### 2.2. Experimental Methods and Procedures

This study adopted a high-temperature oxidation method to achieve two objectives: the removal of sizing agents and the roughening of the carbon fiber surface. Oxidation temperatures were systematically varied at 300 °C, 400 °C, 500 °C, and 600 °C, with a consistent heating duration of 30 min. Following the oxidation process, the carbon fibers were immersed in acetone solution for 30 min and then rinsed with deionized water and dried. To analyze the effect of the oxidation process on the surface morphology, scanning electron microscopy (SEM) and X-ray photoelectron spectroscopy (XPS) were utilized. Based on the results, carbon fibers exhibiting optimal surface properties were selected for Ni electroplating. The electroplating process employed NiSO_4_·6H_2_O as the Ni source, H_3_BO_3_ as a pH buffer, and NiCl_2_·6H_2_O and CH_3_(CH_2_)_11_OSO_3_Na as additives to promote coating quality. Ni plating on the carbon fiber surface was conducted utilizing either a single variable voltage or current, with the plating time fixed at 5 min. The temperature of the plating solution was maintained at room temperature (25 °C) throughout the experiment. The specific composition of the bath is shown in Table 2.

### 2.3. Testing and Characterization

In this study, ESCALAB 250Xi X-ray photoelectron spectroscopy was selected. In this experiment, the test ray source was Al Kα, the incidence angle was 90 degrees, the test depth was 6–10 nm, the energy resolution was 0.6 eV, and the scanning step size was 0.05 eV. After the completion of the test, XPS PEAK 4.1 software was used to carry out the peaking fitting process for the carbon spectrum. The wave number range of 1000~4000 cm^−1^, the scanning time of 64, and the resolution of 2 cm^−1^ were measured using a Nicolet i S50 infrared spectrometer. The JSM-IT500 scanning electron microscope (Japan Electronics Co., LTD, Akishima, Japan) was used, and its voltage was 20 kV, its current was 40 mA, and its resolution was between 1 μm and 1 nm. The surface of the carbon fibers was characterized. The structure of the modified carbon fibers was characterized using a Lab RAM HR Raman spectrometer produced by HORIBA, Longjumeau, France. The wavelength of the laser was 532 nm, and the scanning wave number was 500~2500 cm^−1^.

## 3. Results and Discussion

### 3.1. Surface Modification of the Carbon Fibers

**Figure 1:**
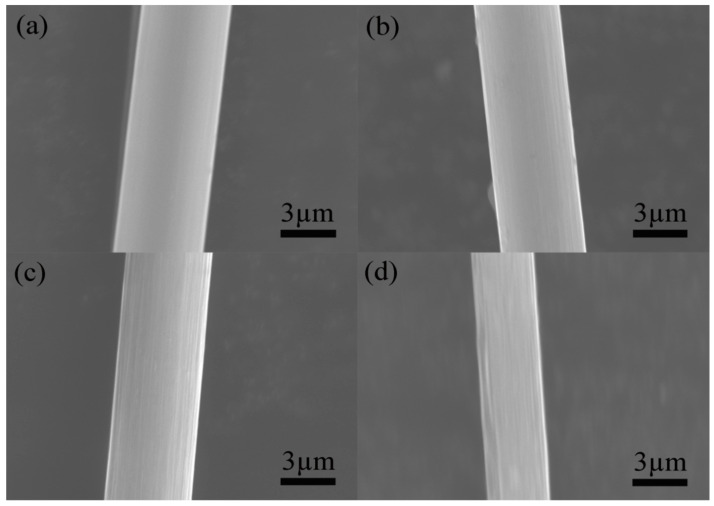
Morphology of carbon fibers under different temperature treatments: (**a**) 300 °C; (**b**) 400 °C; (**c**) 500 °C; (**d**) 600 °C.

Carbon fiber materials, due to their lack of catalytic activity for various chemical plating reactions, are recognized as challenging substrates for plating processes [17,18,19]. To facilitate successful chemical plating on carbon fiber surfaces, specific pretreatment modifications are required to introduce the necessary catalytic activity. Air oxidation treatment is a commonly employed method; however, optimizing the treatment temperature is crucial. Insufficient heating during air oxidation may result in incomplete removal of the sizing agents and oil contaminants from the fiber surface. Conversely, excessive burning temperatures can lead to excessive oxidation of the carbon fibers, causing significant mass loss and reducing their reinforcement effect, ultimately compromising the performance of the composite material [20]. To determine the optimal oxidation temperature, a series of heat treatment processes were conducted at various temperatures, maintaining a consistent heating duration of 30 min, as established in the existing literature. The effect of the temperature difference on the surface morphology of the carbon fibers was analyzed with SEM. As depicted in Figure 1a, burning treatment at 300 °C did not induce significant surface roughening, and residual organic adhesive remained evident on the short carbon fibers. Increasing the temperature to 400 °C resulted in more pronounced grooves on the fiber surface (Figure 1b). A further increase in the oxidation temperature to 500 °C (Figure 1c) yielded surface grooves and a deepened texture, with the original fiber bundles arranged axially, culminating in a rough and uneven surface morphology. At 600 °C (Figure 1d), however, excessive burning of the carbon fiber surface was observed, leading to significant mass loss and the loss of a modification effect.

**Figure 2:**
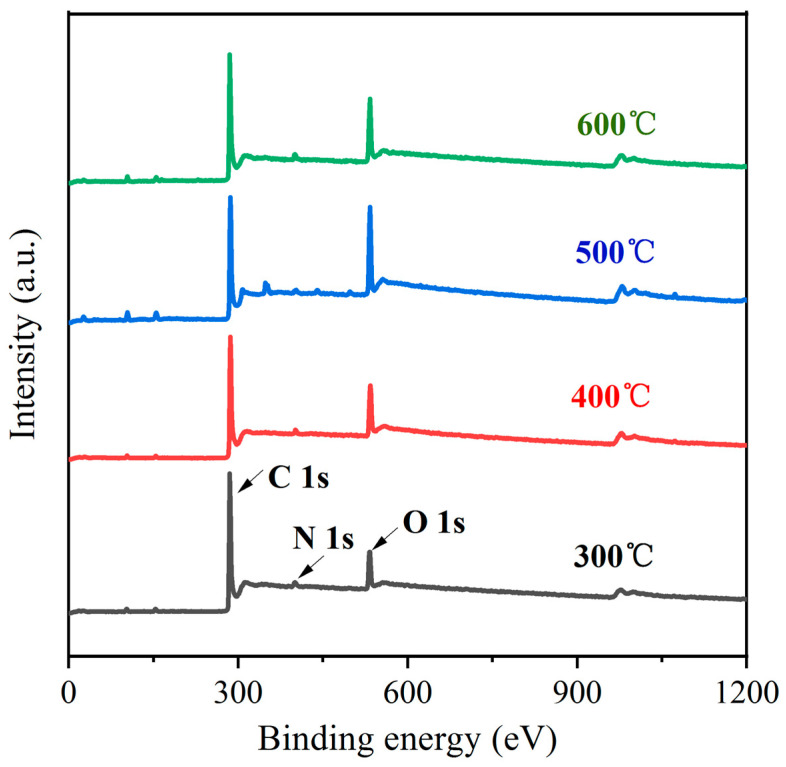
XPS of carbon fibers at different oxidation temperatures.

Surface changes to carbon fibers, particularly a change in the oxygen-containing functional groups, play a critical role in enhancing their efficacy in various applications. XPS offers valuable insights into the quantitative analysis of these functional groups before and after oxidation. Figure 2 presents the XPS spectra of carbon fibers subjected to oxidation at varying temperatures. Specifically, all the samples exhibit prominent C1s and O1s peaks. The presence of the O1s peak in oxidized carbon fibers is indicative of the incorporation of oxygen-containing functional groups during the oxidation process. In addition, the intensity of the O1s peak for the carbon fibers oxidized at 500 °C surpasses that observed at other temperatures, suggesting that this specific condition yields the highest concentration of oxygen-containing functional groups on the fiber surface.

To visually observe the effect of the oxidation temperature on the prevalence of oxygen-containing functional groups on the carbon fiber surface, Table 3 presents the elemental content of C1s and O1s before and after oxidation, along with the O1s/C1s atomic percentage. As presented in Table 3, the O1s/C1s ratio exhibits a significant increase from 15.23% at 300 °C to 34.92% after oxidation at 500 °C. This finding suggests a significant rise in the number of active functional groups on the surface of the oxidized fibers, indicating that the content of oxygen-containing functional groups reaches its maximum at 500 °C. Thereafter, the O1s/C1s ratio demonstrates a decline with further increases in the oxidation temperature.

**Figure 3:**
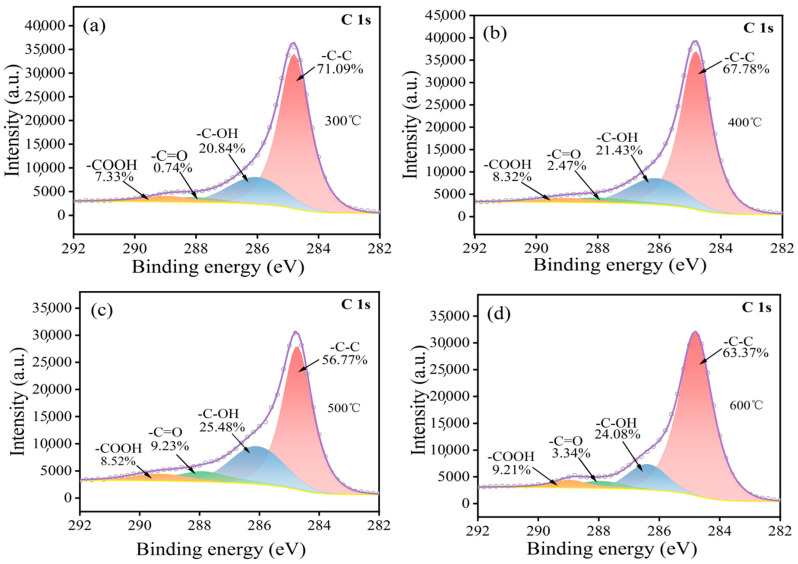
Peak spectrum of C1s before and after oxidation treatment of carbon fibers at (**a**) 300 °C; (**b**) 400 °C; (**c**) 500 °C; (**d**) 600 °C.

Figure 3 presents the C1s peak spectra obtained from the surface of carbon fibers subjected to oxidation at varying temperatures. Analysis of these spectra, based on the established binding energy values for different carbon valence states, indicates the presence of hydroxyl (-OH), carboxyl (-COOH), and carbonyl (-C=O) functional groups as the primary surface functionalities [21,22,23,24]. With increasing temperature, the organic glue on the surface undergoes decomposition, releasing water. This dehydration reaction preferentially occurs for -OH groups, initially leading to an increase in their surface concentration. As the organic glue is progressively removed at temperatures exceeding 300 °C, the introduction of oxygen-containing functional groups through oxidation becomes dominant, resulting in a net increase in their surface population. Figure 3c illustrates that the abundance of these functional groups reaches a maximum at 500 °C. Specifically, the -C=O content also peaks at this temperature. Barinov et al. [25]. have proposed a mechanism for the generation of -C=O groups, suggesting that the formation of specific functional groups on the carbon fiber surface is influenced by the distance between adjacent defective carbon atoms. For instance, when the C-C distance in ethers is small, the formation of vacancies between neighboring carbon atoms is unlikely. Conversely, the probability of -C=O group formation is enhanced. However, as the temperature continues to rise and reaches 600 °C, significant mass loss occurs, leading to a decline in the number of oxygen-containing functional groups on the carbon fiber surface (Figure 3d). Based on this comparative XPS analysis, two key conclusions can be drawn: (1) High-temperature oxidation treatment effectively introduces -C=O and -COOH groups onto the carbon fiber surface, while also increasing the -OH content. (2) Oxidation treatment at 500 °C yields the highest concentration of oxygen-containing functional groups, such as -OH and -C=O, on the carbon fiber surface.

**Figure 4:**
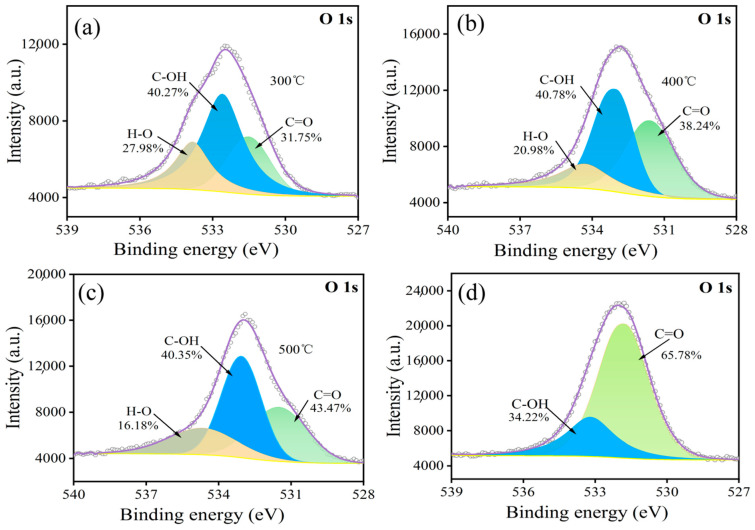
Peak spectrum of O1s before and after oxidation treatment of carbon fibers at (**a**) 300 °C; (**b**) 400 °C; (**c**) 500 °C; (**d**) 600 °C.

Figure 4 presents the O1s peak spectra obtained from the surface of carbon fibers subjected to oxidation at varying temperatures. Figure 4 presents the C1s peak spectra obtained from the surface of carbon fibers subjected to oxidation at varying temperatures. Analysis of these spectra, based on the established binding energy values for different oxygen valence states, indicates the presence of C-OH, C=O, and H-O functional groups as the primary surface functionalities. With an increase in temperature, the content of C=O groups keeps increasing, while the content of H-O groups keeps decreasing, as shown in Figure 4a–c. When the temperature exceeds 600 °C, as shown in Figure 4d, H-O groups disappear, and the content of C=O groups increases to 65.78%. This is because the number and type of oxygen-containing functional groups on the surface of the carbon fiber change with an increase in the heating temperature. The carbonyl group content (C=O) is continuously increased, and the conversion of H-O into water means the H is consumed.

**Figure 5:**
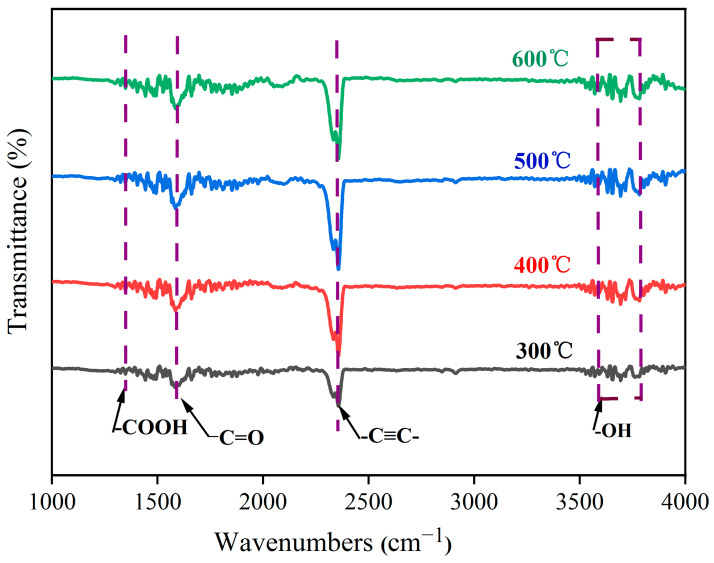
Infrared absorption spectra of carbon fibers before and after oxidation treatment.

While XPS offers valuable insights into the surface composition of fibers to a depth of several nanometers, it is crucial to complement this information with an analysis method capable of probing deeper surface layers. Infrared absorption spectroscopy, with its ability to qualitatively analyze depths of 2–3 μm, serves as a suitable complementary method. Therefore, this study presents the FT-IR spectra of carbon fibers oxidized at various temperatures. As illustrated in Figure 5, all the samples exhibit absorption peaks near 3640 cm^−1^ and 1630 cm^−1^. The strong and broad peak observed near 3640 cm^−1^ can be attributed to the stretching vibration of -OH groups [26,27,28]. Specifically, significant changes occur in both the -OH absorption peak and the -C=O absorption peak near 1630 cm^−1^ with increasing burning temperature. Following oxidation treatment, the intensity of the -OH absorption peak initially increases, reaching a maximum before declining with further temperature increases. This trend reflects the behavior observed for the -C=O absorption peak [29,30,31], which exhibits its highest intensity after oxidation at 500 °C. Based on this analysis, it can be concluded that oxidized carbon fibers possess a significant number of oxygen-containing functional groups, including -OH and -C=O. When -OH and -C=O are simultaneously attached to the same carbon atom, -COOH groups are formed. These findings from infrared absorption spectroscopy align with the XPS results, demonstrating that in a specific temperature range, the content of active oxygen-containing groups (-OH and -C=O) increases with a rising burning temperature, reaching a maximum concentration before decreasing. Combining the results of the XPS and FT-IR analyses, it is inferred that the surface of carbon fibers oxidized in air at 500 °C exhibits the highest concentration of active functional groups. At burning temperatures exceeding 500 °C, the oxidation process becomes more severe, leading to a gradual loss of the strengthening effect of the carbon fibers. Therefore, a burning temperature of approximately 500 °C is selected, ensuring complete removal of the sizing agents from the fiber surface while maximizing the content of oxygen-containing functional groups and minimizing carbon fiber mass loss.

**Figure 6:**
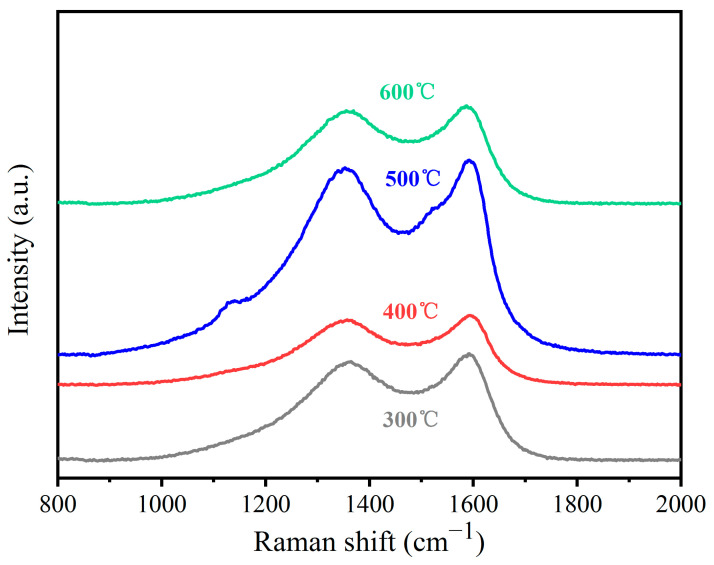
Raman spectrum of carbon fiber after oxidation treatment.

Raman spectroscopy is used to analyze the graphitization degree and ordering degree of the surface crystalline structure of carbon fiber at different oxidation temperatures, and the results are shown in Figure 6. The figure shows that the Raman spectra of carbon fiber treated with different oxidation temperatures have two distinct characteristic peaks, namely a D peak and a G peak. Among them, peak D represents disordered carbon, reflecting the degree of disorder of the crystalline structure, usually appearing in the range of 1340~1350 cm^−1^. Peak G represents the first-order scattering E2g vibration mode of the sp^2^ carbon atoms in the graphite structure, reflecting the integrity of the sp2 hybrid bond structure, which usually appears in the range of 1590~1600 cm^−1^ [32,33]. The degree of the graphitization order of carbon materials is usually judged by the ratio R (I_D_/I_G_) of peak D to peak G. The larger the value of R, the higher the degree of disorder of carbon materials. According to the calculation of the peak strength, the R values for 300 °C, 400 °C, 500 °C, and 600 °C are 0.925, 0.937, 0.962, and 0.950, respectively, indicating that the surface graphitization degree and number of functional groups of carbon fiber treated at 500 °C are the highest.

**Figure 7:**
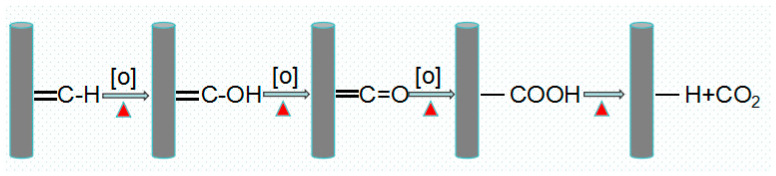
Chemical reactions during combustion process of carbon fibers.

Through a combined analysis of XPS, FT-IR, and Raman data, the oxidative process for carbon fibers can be explained (Figure 7) [34,35]. Part of the active carbon on the surface of the carbon fibers reacts with [O] to produce -OH groups, while many surface -C=O groups are oxidized into -COOH groups, and a small portion are oxidized into CO_2_. In this process, due to the low enthalpy of -OH group generation, the formation of -OH groups is the main reaction, resulting in an increase in the -OH group content. As the temperature rises, the reaction progresses, leading to the oxidation of a significant portion of -OH groups into -COOH groups. At this juncture, the generation and subsequent oxidation of -OH groups become competing reactions, leading to a gradual increase in the -COOH group content. In addition, active carbon directly reacts with free oxygen to generate -C=O groups. However, continued oxidation of these -C=O groups results in the formation of either -COOH groups or CO_2_, representing a competing reaction pathway. This finally results in an increase in the -C=O group content. This series of oxidation reactions is highly exothermic, leading to the continuous depletion of carbon atoms in the carbon fibers. This phenomenon is reflected in the rapid increase in carbon fiber burn-off weight observed above 500 °C (Figure 1).

### 3.2. Ni Electrodeposition on Carbon Fiber Surfaces

**Figure 8:**
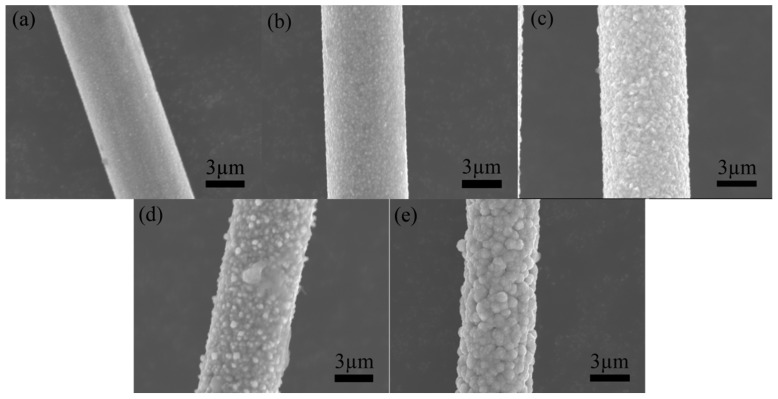
Morphology of Ni electroplating on carbon fiber surfaces at different voltages: (**a**) 5 V; (**b**) 7.5 V; (**c**) 10 V; (**d**) 12.5 V; (**e**) 15 V.

This study employed a single-variable control method to evaluate the effect of a stable voltage on the quality and morphology of Ni-plated carbon fiber coatings. SEM images of the Ni-plated carbon fiber surfaces at various voltages are presented in Figure 8. At a voltage of 5 V (Figure 8a), the quantity of Ni deposited on the carbon fiber surface is minimal due to the low voltage, resulting in incomplete coverage of the fiber surface by the Ni layer. Increasing the voltage to 7.5 V (Figure 8b) yields a smooth, delicate, and uniformly intact Ni-plated layer that tightly encapsulates the carbon fiber. Further voltage increases lead to roughening of the carbon fiber surface and the emergence of granular Ni deposition (Figure 8c,d). At a voltage of 15 V (Figure 8e), the high instantaneous voltage promotes the continuous growth of Ni-deposited atoms at specific points, finally forming spherical Ni-plated layers. The formation mechanism of these spherical Ni layers will be elaborated upon in the following section.

**Figure 9:**
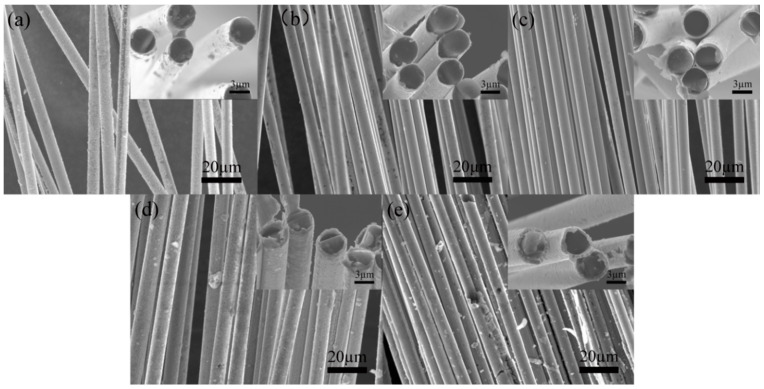
Morphology of Ni electroplating on carbon fiber surfaces at different current intensities: (**a**) 0.2 A; (**b**) 0.4 A; (**c**) 0.6 A; (**d**) 0.8 A; (**e**) 1.0 A.

The current density plays a critical role in the electroplating process, significantly affecting the deposition rate and coating thickness. This study evaluates the effect of a constant current on the morphology of Ni-plated carbon fibers. The SEM images in Figure 9 illustrate the morphological evolution of the Ni coating under different current conditions. At a low current of 0.2 A (Figure 9a), the coating exhibits an insufficient thickness due to the low current density, resulting in incomplete surface coverage. Increasing the current to 0.4 A (Figure 9b) leads to a thicker coating, with Ni atoms primarily covering the carbon fiber surface. At 0.6 A (Figure 9c), the Ni layer completely and uniformly encapsulates the carbon fiber, exhibiting a smooth and dense morphology. However, further increasing the current to 0.8 A (Figure 9d) induces surface roughness and the emergence of cellular Ni precipitates. At 1 A (Figure 9e), uniform Ni plating becomes unattainable, with localized Ni atom precipitation causing metallic adhesion between carbon fibers and generating significant coating defects and fiber damage. Furthermore, increased current densities promote side reactions such as hydrogen evolution, leading to H_2_ adsorption on the coating, which can induce bubbling and peeling.

To further understand the relationship between coating thickness, deposition rate, and time, this study determined the deposition rate V (µm/h) according to the ASTM B733 standard. In Equation (1), w_0_ (mg) and w_1_ (mg) represent the weights before and after deposition, respectively; ρ (g/cm^3^) denotes the density of the Ni layer, selected as 8.9 g/cm^3^; A (cm^2^) expresses the surface area of the test sample; and t (h) indicates the plating time [36,37].

(1) $$V=\frac{\left(w_{1}-w_{0}\right)}{\rho \times A\times t}\times 10^{4}$$

The deposition rate V (µm/h) can also be expressed by Equation (2), where d (µm) represents the coating thickness.

(2) $$V=\frac{d}{t}$$

Combining Equations (1) and (2), the coating calculation equation can be obtained as follows:

(3) $$d=\frac{\left(w_{1}-w_{0}\right)}{\rho \times A}\times 10^{4}$$

**Figure 10:**
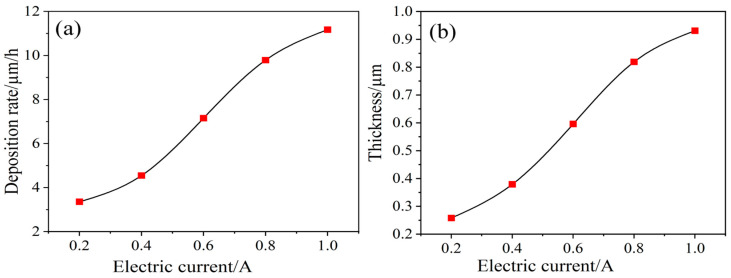
Deposition rate and coating thickness of carbon fibers at different current intensities: (**a**) deposition rate; (**b**) coating thickness.

Utilizing the above equations, the deposition rate and coating thickness at different currents can be obtained (Figure 10).

### 3.3. Mechanism of Ni Electrodeposition on Carbon Fiber Surfaces

The efficacy of carbon fibers in reducing metal ions hinges on the presence of surface functional groups conducive to the reduction process. Specifically, the presence of reducible functional groups facilitates the decrease in the valence state of metal ions. Therefore, an increased concentration of such functional groups on the carbon fiber surface enhances the probability of nickel ion reduction, reducing the adsorption capacity. From the standpoint of redox reactions, the active functional groups on the carbon fiber surface engage in chemical reactions with Ni^2+^ ions in the solution. The reaction equations are given as follows [38,39]:

-C-H + Ni^2+^ + H_2_O → -C-OH + Ni + 2H^+^ (4)

2-C-OH + Ni^2+^ → 2-C=O + Ni + 2H^+^ (5)

2-C=O + Ni^2+^ + 2H_2_O → 2-COOH + Ni + 2H^+^ (6)

**Figure 11:**
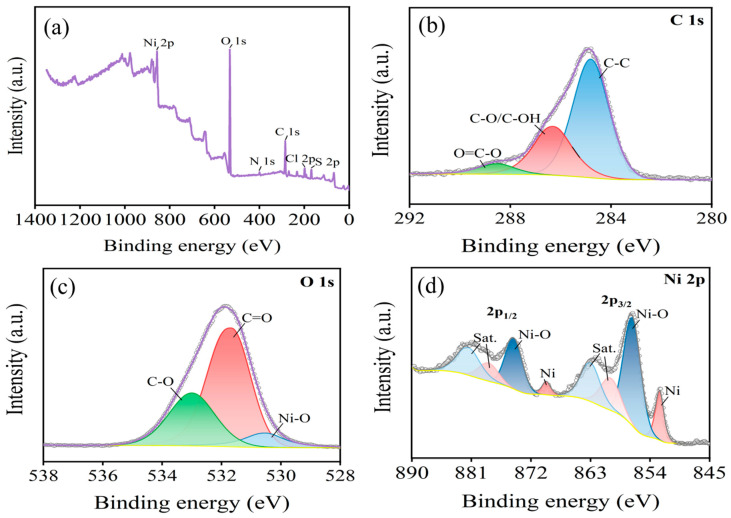
XPS spectra of Ni electrodeposition on carbon fiber surfaces: (**a**) full spectrum of Ni electrodeposition; (**b**) C1s spectrum; (**c**) O1s spectrum; (**d**) Ni2p spectrum.

The aforementioned reaction equations suggest that the -C-H, -OH, and -C=O functional groups present on the surface of carbon fibers play a crucial role in the reduction of Ni^2+^ ions in the solution. While the -C-H content is primarily determined by the specific production process of the carbon fibers, the -OH and -C=O content can be modulated by adjusting the oxidation temperature. Specifically, the presence of these functional groups enhances the activity of the carbon fiber surface, facilitating the reduction of Ni^2+^ ions from the solution into metallic Ni. To further illustrate the elemental composition and distribution of functional groups on the Ni-electrodeposited carbon fiber surface, XPS analysis was conducted on the Ni-plated layer (Figure 11).

Figure 11a presents the XPS spectrum of the Ni-plated carbon fibers, indicating the presence of Ni, O, and C elements on the fiber surface. The presence of O suggests a significant abundance of oxygen-containing functional groups in the Ni-plated layer, potentially enhancing the surface activity of the coating. Conversely, the low C content implies the formation of a dense Ni-plated layer on the carbon fiber surface. Further analysis in Figure 11b indicates that C in the coating primarily exists in the form of functional groups such as C-OH and C=O. These groups are known to facilitate further Ni deposition on the surface. It can be seen from Figure 11c that oxygen mainly exists in the form of C=O, C-O, and Ni-O. Figure 11d displays the Ni2p peak, characterized by two primary peaks, 2p_3/2_ and 2p_1/2_, each accompanied by corresponding satellite peaks located at 860 eV and 878 eV, respectively [40,41]. This spectral signature confirms the presence of Ni^2+^ in the coating. This observation suggests that Ni on the coating’s outer layer primarily exists as elemental Ni and NiO, indicating the oxidation of surface Ni upon air exposure. During the plating process, Ni^2+^ ions in the plating solution migrate towards the carbon fiber and deposit around its surface. The abundance of oxygen-containing functional groups on the treated fiber surface facilitates the formation of Ni-C-O bonds between Ni deposition and these polar groups, thereby promoting strong adhesion between the Ni coating and the fiber. Ni deposition on the cathode fiber retains its electronegative character, enabling it to continuously offer electrons. Therefore, Ni^2+^ ions in the plating solution are attracted to the surface, where they undergo reduction into metallic Ni and deposition. This process leads to a gradual increase in the coating thickness as the plating time is extended.

**Figure 12:**
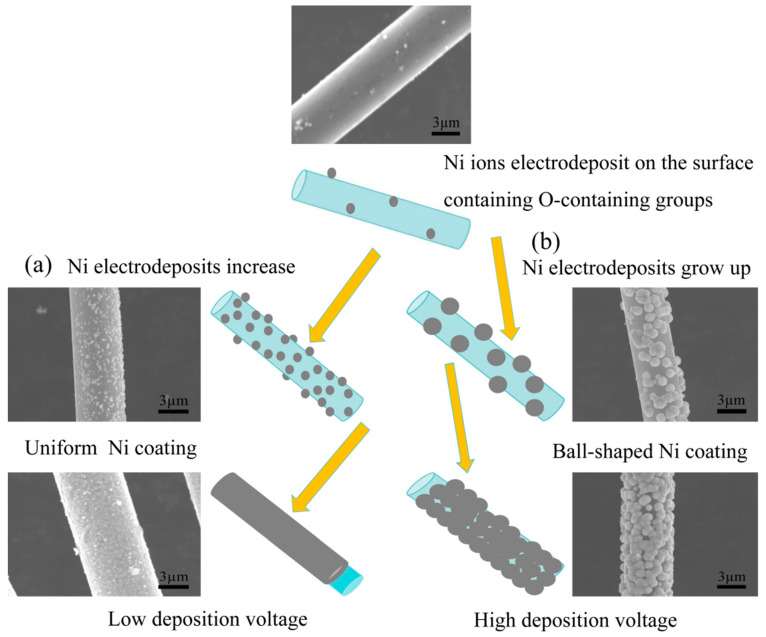
Schematic of Ni deposition with different morphologies on carbon fiber surfaces. (**a**) low deposition voltage (**b**) High deposition voltage.

The reduction of Ni^2+^ ions during electrodeposition onto carbon fibers is a process influenced by multiple factors beyond the applied deposition voltage. The chemical state of the fiber surface, specifically the presence of oxygen-containing functional groups, plays a crucial role in facilitating the reduction process. These functional groups, such as -OH and -COOH, act as active sites with a lower potential, thereby promoting electrochemical reactions [42,43]. Upon the application of an electric potential, Ni^2+^ ions are preferentially reduced at these active sites due to their lower energy barrier. This initial reduction leads to the adsorption of metallic Ni at these locations. However, due to the limited number of active functional groups, the Ni particles on the fiber surface remain sparse at this stage, as depicted in the top image in Figure 12. This stage of electrocrystallization follows instantaneous nucleation and a three-dimensional growth mode. With the sustained application of a low deposition potential (near the equilibrium potential), the rate of nucleation generation and dissolution remain comparable, resulting in a weak reduction of Ni^2+^ into metallic Ni. As the deposition potential increases, additional functional groups participate in the reduction reaction, leading to a higher density of reduction points. However, with further increases in the deposition potential, the effect of the functional groups becomes less significant. The process becomes dominated by the initial nucleation and growth driven by the applied potential, resulting in a uniform and smooth deposition layer on the fiber surface (Figure 12a). In contrast, when a high deposition potential is applied instantaneously, the rapid reduction of Ni crystals at the active sites enhances the local conductivity. This leads to the preferential reduction of Ni^2+^ ions at these points, causing the Ni crystals to grow larger. This self-growth is further amplified by the electrodeposition tip effect, characterized by a faster reduction rate of the metal ions at the tip, finally leading to the formation of a spherical morphology (Figure 12b). Therefore, the reduction of Ni ions during Ni electrodeposition is not solely driven by the applied potential. Instead, it arises from an additive effect between the applied potential and the reducing action of the active functional groups on the carbon fiber surface. These functional groups promote the preferential reduction of Ni^2+^ ions at specific locations, influencing the morphology and distribution of the deposited Ni layer.

## 4. Conclusions

(1) High-temperature oxidation has been demonstrated to be an effective method for modifying the surface of carbon fibers, leading to improved adhesion properties. This study has presented that a temperature of 500 °C exhibits the highest concentration of functional groups such as -OH, -C=O, and -COOH, resulting in the most effective modification. It is crucial to note that exceeding this temperature threshold leads to a significant loss of carbon fiber mass, thereby compromising its strengthening capabilities.
(2) In the process of the heating and oxidation of carbon fiber, its surface functional groups constantly change. At a low heating temperature, the surface is mainly dominated by -OH groups and -C=O groups. With an increase in temperature, a large number of -OH groups are oxidized into -COOH groups, and the continuous oxidation of some -C=O groups leads to the formation of -COOH groups or CO_2_. This causes loss of the C element on the carbon fiber surface.
(3) Further modification through Ni electrodeposition under stable voltage conditions indicates that a low voltage yields a Ni-plated layer on the carbon fibers that is both smooth and uniform. Application of an insufficient voltage results in incomplete Ni deposition, while excessive voltage leads to surface roughness and the undesirable presence of particle deposits in the plated layer. Specifically, at a high voltage (15 V), a spherical Ni-plated layer forms on the carbon fiber surface.

## Figures and Tables

**Table 1:** Composition and performance indicators of carbon fiber.

Carbon Content (%)	Tensile Strength (GPa)	Modulus (GPa)	Density (g/cm^3^)	Diameter (µm)	Elongation (%)
≥93	≥3.0	210–240	1.77	6–8	≥1.5

**Table 2:** Composition of nickel plating solution.

NiSO_4_·6H_2_O	NiCl_2_·6H_2_O	H_3_BO_3_	(CH_3_(CH_2_)_11_OSO_3_Na	Temperature	Time
260 g/L	60 g/L	40 g/L	0.1 g/L	25 °C	5 min

**Table 3:** Surface composition analysis of carbon fibers after oxidation at different temperatures.

Sample Name	C1s/%	O1s/%	O1s/C1s/%
300 °C CFs	86.78	13.22	15.23
400 °C CFs	84.40	15.60	18.48
500 °C CFs	74.12	25.88	34.92
600 °C CFs	81.38	18.62	22.88

## Data Availability

The data that support the findings of this study are available from the corresponding author upon reasonable request.
